# MHNfs: Prompting In-Context Bioactivity Predictions for Low-Data Drug Discovery

**DOI:** 10.1021/acs.jcim.4c02373

**Published:** 2025-04-30

**Authors:** Johannes Schimunek, Sohvi Luukkonen, Günter Klambauer

**Affiliations:** ELLIS Unit Linz and LIT AI Lab, Institute for Machine Learning, Johannes Kepler University Linz, A-4040 Linz, Austria

## Abstract

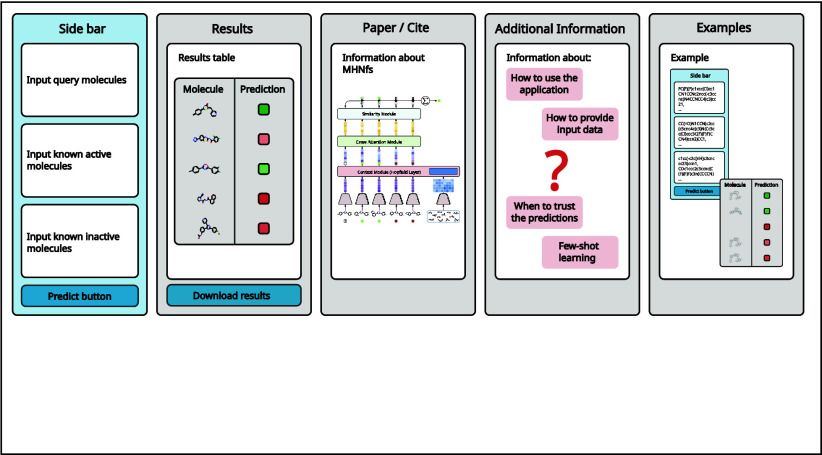

Today’s drug discovery increasingly relies on computational and machine learning approaches to identify novel candidates, yet data scarcity remains a significant challenge. To address this limitation, we present *MHNfs*, an application specifically designed to predict molecular activity in low-data scenarios. At its core, *MHNfs* leverages a state-of-the-art few-shot activity prediction model, named MHNfs, which has demonstrated strong performance across a large set of prediction tasks in the benchmark data set FS-Mol. The application features an intuitive interface that enables users to prompt the model for precise activity predictions based on a small number of known active and inactive molecules, akin to interactive interfaces for large language models. To evaluate its efficacy, we simulate real-world scenarios by recasting PubChem bioassays as few-shot prediction tasks. *MHNfs* offers a streamlined and accessible solution for deploying advanced few-shot learning models, providing a valuable tool for accelerating drug discovery.

## Introduction

### Bioactivity Prediction Models Are Indispensable Tools in Modern Drug Discovery Projects^2−5^

The process of identifying new drug candidates is fraught with challenges: it is inherently complex, expensive, time-consuming, and prone to failure.^3,6^ These difficulties arise from the intricate nature of biological systems,^7^ the vast and largely unexplored chemical space of potential drug candidates,^8−11^ and the high costs associated with experimental validation and clinical trials.^3,12^ To improve the efficiency and cost-effectiveness of this process, machine learning (ML) methods have been increasingly adopted across various stages of the drug discovery pipeline,^7,13^ demonstrating the ability to reduce both time and monetary investment.^14,15^ Among these methods, bioactivity prediction models, which map molecular structure to bioactivity using known data, have emerged as key enablers.^16^ These models are integral to large-scale virtual screening efforts,^17,18^ enabling researchers to efficiently evaluate vast numbers of molecules. Today, a wide variety of such models exist, differing in architecture, training schemes, implementation, and application scope (see Table 1, Model Card1).

**Table 1:** Model Card^1^ - *MHNfs* on HuggingFace

**Model Details**	**Metrics, Training and Evaluation Data**
• Developed by researchers at the Johannes Kepler University Linz, 2023, v1	• Training and evaluation data: FS-Mol
• Embedding-based few-shot method based on Modern Hopfield Networks (Transformer architecture)	• External evaluation data: PubChem assays
• Deployed on HuggingFace: https://huggingface.co/spaces/ml-jku/mhnfs	• Reported metrics:
	– FS-Mol: AUC, ΔAUC-PR
	– PubChem: AUC, ΔAUC-PR, MCC, BACC, and BEDROC
**Intended Use**	**Caveats and Recommendations**
• Intended to be used to predict bioactivities for targets similar to the targets included in the FS-Mol benchmark	• Use model for oxidoreductases, kinases, hydrolases, lysases, isomerases, ligases, and translocases
• User input: Known actives and inactives, and molecules to be predicted (SMILES)	• For other targets, check whether similar targets are included in the FS-Mol training data: https://github.com/microsoft/FS-Mol/blob/main/datasets/targets/target_info.csv
• Model output: Bioactivity table for requested molecules	
**Factors**	
• The performance of the model depends on the target family.	
• Evaluated target families: Oxidoreductases, kinases, hydrolases, lysases, isomerases, ligases, translocases	

### The Landscape of ML-Based Activity Prediction Models and Available Software

Machine learning models for activity prediction come in various forms, including descriptor-based multilayer perceptrons,^17,19^ Random Forests (RFs),^20−23^ gradient-boosting methods,^24,25^ as well as graph neural networks.^26^ To support researchers, several open source software packages^27−31^ provide tools that cover the entire bioactivity prediction pipeline, from molecular data preprocessing to model development and evaluation. Although ML- and deep learning-based bioactivity models and their software are widely used in drug discovery, they are typically tailored to specific tasks that were seen during training. Adapting them to new tasks often requires retraining, which usually demands substantial data, limiting their use in data-scarce scenarios.

### Few-Shot Learning Methods for Low-Data Drug Discovery

Drug discovery projects often start with only a few known molecules,^18,32,33^ whereas standard bioactivity prediction models require large data sets for training.^17,19,34−36^ To address this gap, few-shot learning methods have been developed for low-data scenarios.^37−41^ Among them, MHNfs(42) represents the state-of-the-art that has been trained and evaluated on the FS-Mol benchmark data set.^32^ When provided with a so-called support set of a few active and inactive molecules, MHNfs predicts the molecular activity of query molecules by comparing learned molecule representations and weighting the support set’s labels based on similarity. Training few-shot learning models is resource-intensive and requires expert knowledge, and their use typically demands programming skills and familiarity with deep learning frameworks, limiting their adoption in chemoinformatics and drug discovery projects. Despite recent advancements in accuracy^42^ and practical utility,^43^ these models remain largely inaccessible to many researchers. However, like large language models (LLMs), they could become more accessible through simple and interactive interfaces. Through this work, MHNfs is now made user-friendly via an interactive interface that allows researchers to perform activity predictions with minimal technical expertise, akin to how LLMs are employed for diverse tasks (see Figure 2).

### Prompting-Based Activity Predictions for Low-Data Scenarios

We provide an application that enables the use of the MHNfs model through a simple, interactive user interface based on input prompts. Specifically, we provide a Streamlit application,^44^ hosted on HuggingFace^45^ at https://huggingface.co/spaces/ml-jku/mhnfs. This interface allows users to perform activity predictions for any target, as long as some active and inactive molecules are known. To do so, the user needs to provide three input prompts, which are a) the molecules of interest for which activity predictions are requested, b) the known active molecules, and c) the known inactive molecules. Conditioned on the input prompts, MHNfs computes activity predictions, which are then presented to the user in an accessible and user-friendly format.

## Problem Setting and Summary

Few-shot learning in drug discovery addresses the challenge of building bioactivity models using only a small number of molecules with known bioactivities. These bioactivity values may represent activity in a bioassay or against a specific drug target. The available data, referred to as the *support set*, comprise molecular structures paired with binary bioactivity labels. Few-shot models utilize the support set to predict bioactivity for *query molecules*—those with unknown labels. Recent advancements in few-shot activity prediction methods^32,42,43^ have demonstrated good performance, effectively identifying meaningful patterns and providing reasonable predictions even in very low-data scenarios, and hold great potential to accelerate drug discovery. However, their adoption is limited due to accessibility barriers. In this work, we address this limitation by providing a simple, interactive user interface that makes these models accessible to practitioners.

## Method Overview and Application Details

**Figure 1:**
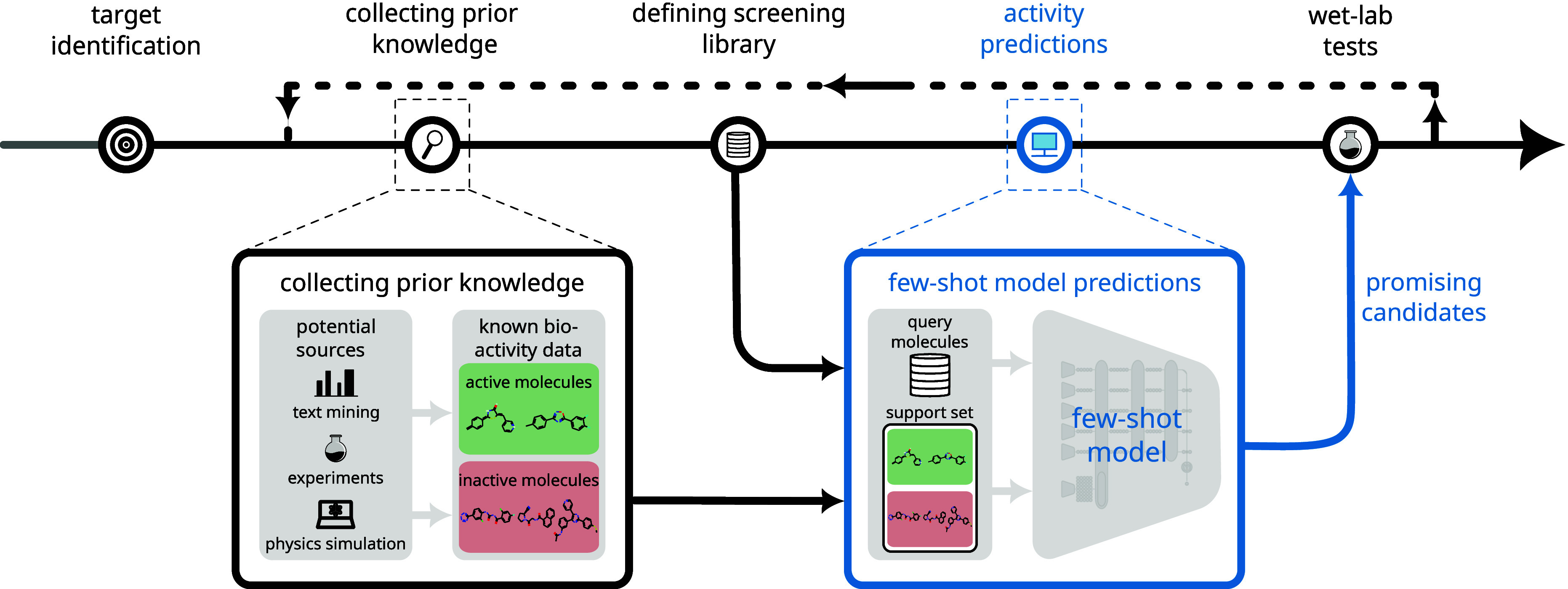
Early stage drug discovery pipeline and the role of *MHNfs*. *MHNfs* is a prompting-based few-shot bioactivity prediction application designed for very early stage virtual compound screening when prior knowledge about bioactivity is minimal.

Figure 1 illustrates the virtual screening process in drug discovery following target identification.^46,47^ Virtual screening is further broken down into three key substeps: collecting prior knowledge, defining the screening library, and predicting bioactivities. Bioactivity models are a cornerstone of virtual screening, typically trained on data sourced from prior experiments, physical simulations, or text mining. However, in the early stages of drug discovery, the amount of available training data is often severely limited. The proposed application, *MHNfs*, is specifically designed to address this low-data challenge, enabling effective bioactivity predictions during the earliest phases of a drug discovery project.

### MHNfs: An Embedding-Based Few-Shot Method for Drug Discovery

The backbone of our application is the MHNfs model.^42^MHNfs consist of three core modules: a) the Context Module, b) the Cross-Attention Module, and c) the Similarity Module.

MHNfs takes two inputs: a support set of known active and inactive molecules and a query set of molecules whose activity needs to be predicted. The model first generates an initial low-level representation of the molecules. This representation is refined through the Context and Cross-Attention Modules. Finally, the updated representations are passed to the Similarity Module, which produces the activity predictions for the query set.

#### Context Module

The Context Module updates input representations by retrieving information from a large set of context molecules, known as the *context set*. This amplifies the covariance structure of the data and removes spurious co-occurrences. The context-enrichment step is conceptually similar to how a large language model (LLM) leverages all available information within its context window (see Section In-Context Learning and Context Enrichment). In MHNfs, the context enrichment is realized with a Modern Hopfield Network^48^ (Transformer architecture^49^). It performs cross-attention between the molecular input representations and the context set. Notably, the representations of query and support-set molecules are updated independently during this process.

#### Cross-Attention Module

The Cross-Attention Module enables information sharing between a query and the support-set representations. This information-sharing step, similar to in-context learning, plays a crucial role in embedding-based few-shot methods.^37^

#### Similarity Module

The Similarity Module computes pairwise similarities between each query molecule and the molecules in the support set. These similarity values are then used as weights in a weighted sum over the support-set labels, ultimately predicting the activity of each query molecule.

### Deployed Model with Preconfigured Context

The MHNfs backbone was trained and evaluated on the FS-Mol benchmark data set,^42^ and its fully trained instance is made accessible through this application. The model is preintegrated with a context set, sampled from the FS-Mol training data, which allows users to perform predictions directly. Consequently, there is no need to retrain the model, adjust its parameters, or manually select a context set; users simply provide the query molecules, along with known active and inactive molecules. For further details on the hyperparameter settings and training procedure, please see Schimunek et al.^42^

### In-Context Learning and Context Enrichment

Following Brown et al.^50^ and Dong et al.,^51^**in-context learning** is a paradigm that allows language models to learn tasks given only a few examples in the form of demonstrations. Specifically, a language model is provided with an optional task instruction, known data, and a query sample, and it generates a prediction for the query. In in-context learning, the term context refers to the task-specific information provided to the model as input, including the optional instruction and the known data used to prompt the model.

Long-context LLMs are large language models equipped with an extended context window, enabling them to retrieve and process information from a large corpus,^52,53^ such as text chunks from documents or conversation histories in chatbots.^54^ This large context window has been shown to significantly enhance performance across various tasks.^55,56^ Notably, for downstream tasks, the long context might contain not only task-specific information but also more general information to enrich the encoded tokens with additional information. We refer to this mechanism as **context enrichment**.

**Figure 2:**
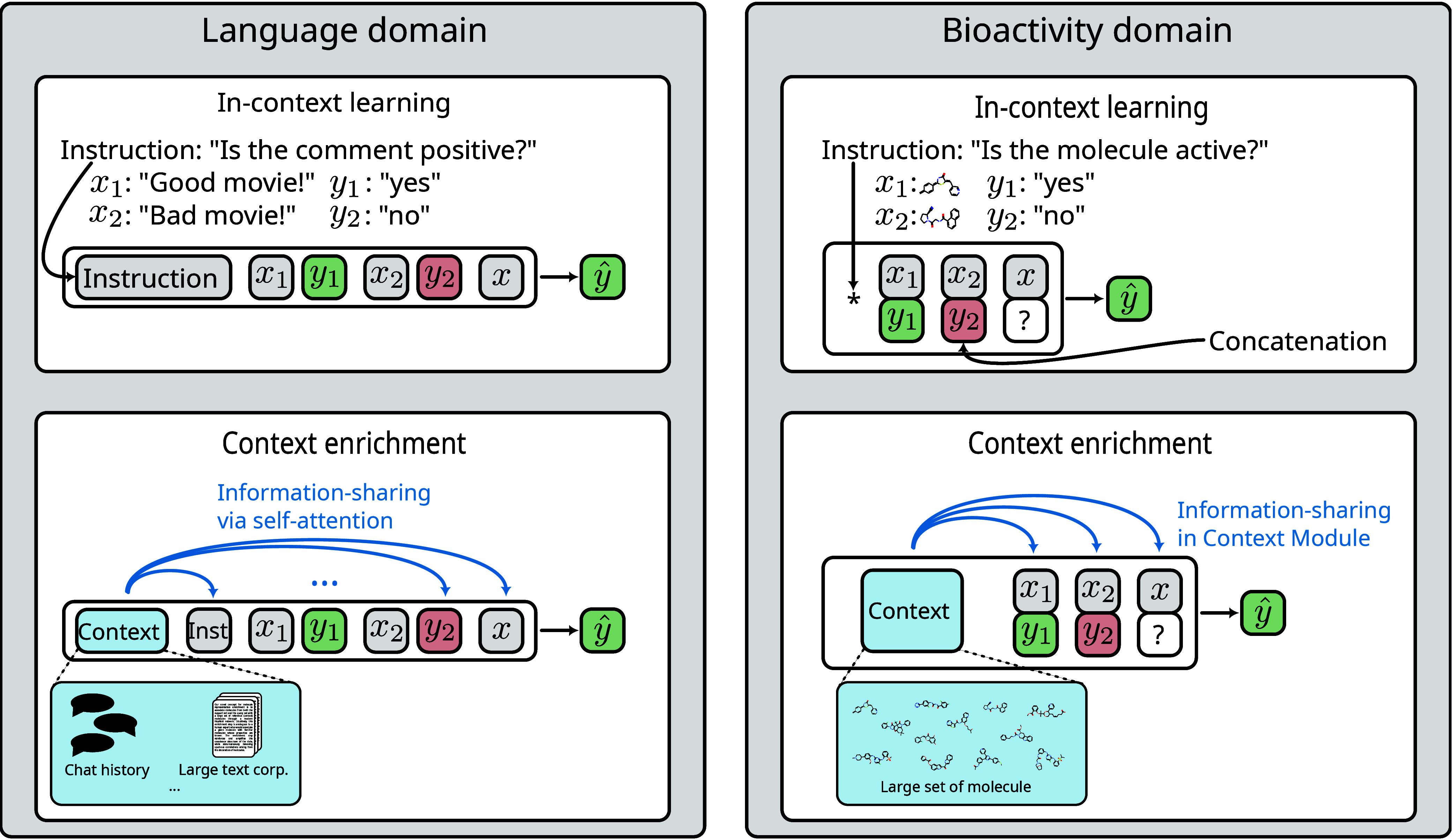
In-context learning and context enrichment. Left: Language domain (adapted from Chen et al.^57^ Copyright The Authors. CC BY 4.0). In in-context learning, few-shot tasks are solved by providing an instruction together with known labels and a query *x* to the LLM. The context window usually includes wider information, e.g., a chat history or a large text corpus, and shares information via self-attention. Right: Bioactivity domain. Analogously, the model is provided with known data (support set) and a query to output activity. Since the instruction does not change for different tasks, it is omitted (indicated by *). MHNfs includes wider context, here a large set of training molecules, by retrieving information via the Context Module.

For few-shot drug discovery, the in-context learning definition can be easily adopted. In this sense, MHNfs is an in-context learning method (see Figure 2). The model is prompted with a support set and a query molecule. The support set includes the known available data for a task in the form of some active and inactive measured molecules. Since for bioactivity models, the task instruction, i.e., “predict activity” is constant for all tasks, it can be omitted. MHNfs also includes a context-enrichment mechanism in which broader, more general, not task-specific information is used. The Context Module enriches the vector representations of query and support-set molecules with information retrieved from a large set of additional molecules which were included in the training data.

### *MHNfs* HuggingFace Interface

**Figure 3:**
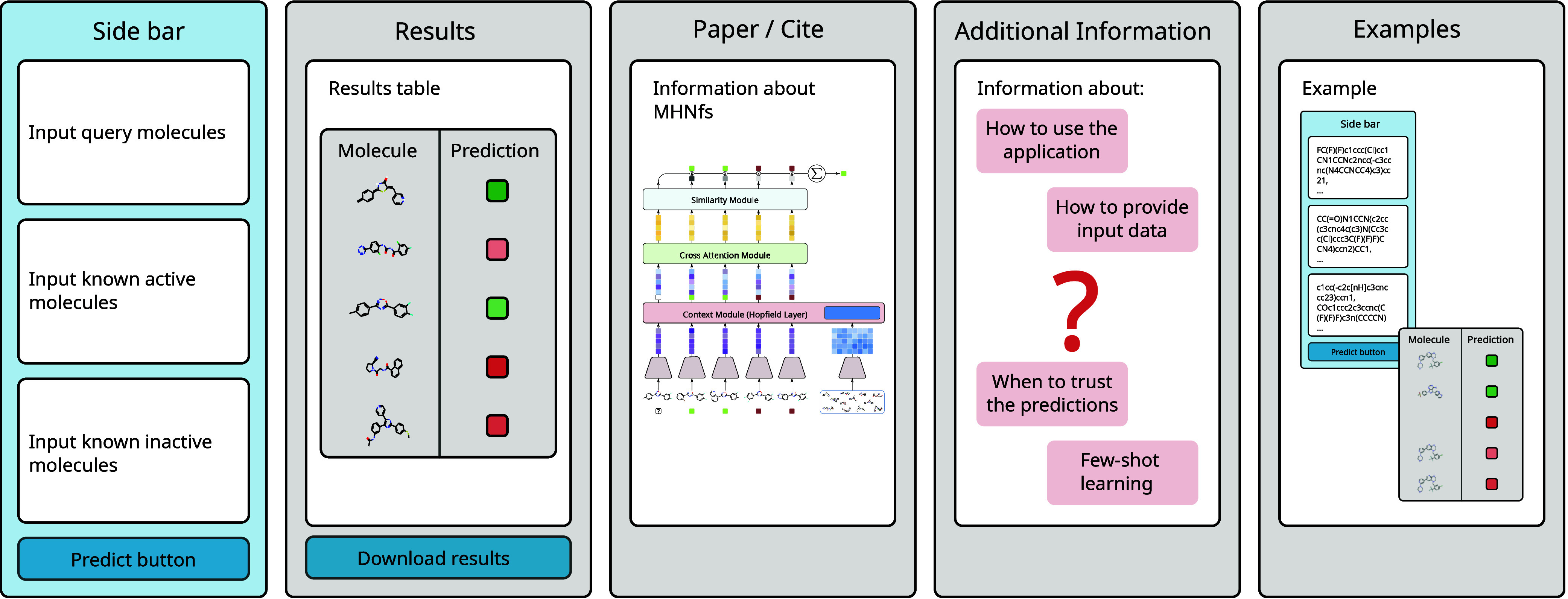
Schematic overview of the Streamlit interface of *MHNfs*. The interface empowers users to input data and execute the pipeline via the sidebar. The main area, highlighted by gray-colored boxes, displays results and additional information. Organized into tabs, represented as separate boxes in the schematic, the main area provides users with an intuitive way to access and explore outputs and supplementary details.

The HuggingFace^45^ application is based on the Streamlit package.^44^ It consists of a sidebar and a main area (Figure 3). The sidebar allows users to prompt the model by providing: a) known active molecules, b) known inactive molecules, and c) a set of molecules to predict. These inputs can be entered through text boxes or uploaded as CSV files, with molecules expected in SMILES notation.

The main area includes four tabs: Predictions, Paper/Cite, Additional Information, and Examples. The **Predictions** tab displays results as a table containing queried molecules and their predicted activities, along with a grid plot showing 2D molecular graphs, SMILES strings, and predicted activities. The **Paper/Cite** section links to the publication describing the backbone model of the application. The **Additional Information** section provides details on few-shot learning, MHNfs, and guidance for using the application, including a *When to trust the predictions* section that emphasizes staying within the training domain for new tasks. The **Examples** tab offers one example task in which the application performs well and another task in which it is not suitable. This design ensures users can easily interact with the application, access relevant information, and understand its capabilities and limitations.

### Model Evaluation

In the original method paper,^42^MHNfs was evaluated on the FS-Mol test set. Here, we perform a new experiment and evaluate the *MHNfs* application on different external experimental setups by recasting assay data from PubChem^58^ as few-shot scenarios. This evaluates the generalization capabilities of MHNfs because the model was originally trained and evaluated on FS-Mol, which is an excerpt from ChEMBL27,^59^ and in this work, the model was further evaluated on data from a different source and unseen protein targets without adjusting any of the model parameters.

#### Review of the FS-Mol Experiments

MHNfs(42) was developed and evaluated using the FS-Mol benchmark data set, which comprises 4,938 training tasks and 157 test tasks derived from assay data in ChEMBL27. ChEMBL27^59^ is a semimanually curated database of bioactivity values of drug-like molecules—with automated steps to improve data quality^60,61^—that contains approximately 2.4 million compounds and over 15 million activity data points, typically sourced from peer-reviewed scientific publications. On the relatively high-quality FS-Mol test set, which is comparable in quality to the training data, MHNfs outperformed other few-shot models, including ADKF-IFT,^41^ the IterRefLSTM-based few-shot model,^37^ Siamese Networks,^62^ Prototypical Networks,^63^ and Property-Aware Relation Networks.^40^ For further details on the FS-Mol test set evaluations and extended results, such as performance across different protein families, please refer to Schimunek et al.^42^

#### Evaluation on External PubChem-Based Data Sets

##### Motivation

As we anticipate the application *MHNfs* may also be used in scenarios with reduced data quality, including increased noise and domain shifts compared to the training data, the motivation is to assess the model’s robustness. Recent studies^37,42^ have indicated that few-shot models can suffer from decreased performance under such conditions. To address this concern, we aim to evaluate MHNfs, without adjusting any of the already trained model parameters, using PubChem-based data,^58^ a more general database with diverse and potentially noisier data. Built from over 1,000 data sources, it contains 119 million molecules and 295 million bioactivities, making it significantly larger and more varied, presenting a challenging but important test of MHNfs’ adaptability.

##### Experimental Setup

From PubChem, all single protein assays linked to a protein not present in the FS-Mol training or validation sets were retrieved by comparing UniProt accession keys. The following filtering steps were applied to the retrieved assay data: a) HTS assays with more than 100,000 data points were removed, b) data points with conflicting bioactivity labels were removed, c) molecules were only kept if the MHNfs’ preprocessing script could handle the SMILES input, and d) targets with less than 50 active and inactive molecules were removed. Note that for d), we only require the data points for evaluation, while the actual training sets, i.e., support sets, are very small with only 8 or 16 data points. From the filtered data, different experimental setups were built by varying the bioactivity prediction task types, the number of known molecules, and the ratio of known actives and inactives. To consider different bioactivity prediction task types, two different data sets are built that differ from each other by the way the measurements were merged and the tasks were built. For the first data set, the *by-target data set*, bioactivity data linked to the same target were merged, resulting in 229 different tasks. For the second data set, the *by-assay data set*, measurements were merged according to their assay membership and only assays with at least 30 active and 30 inactive molecules were kept, resulting in 127 different tasks. To mimic scenarios with a different number of known molecules and different ratios in terms of actives and inactives, we consider two different support-set sizes, i.e., 8 and 16, and different active and inactive ratios: 1 (2) active and 7 (14) inactive molecules (ratio 1:7), 2 (4) active and 6 (12) inactive molecules (ratio 1:3), and 4 (8) active and 4 (8) inactive molecules (ratio 1:1).

##### Methods Compared

We compare MHNfs with an RF baseline trained from scratch on the support-set data. The RF baseline was implemented with sklearn using the default hyperparameters. We consider RFs to be a natural default choice for users who want to train models in scenarios with limited data, given their strong performance on small data sets and ease of use.

##### Metrics

The compared models are evaluated on different metrics: the area under the ROC curve (AUC), the area under the precision-recall curve where the difference to a random classifier is reported (ΔAUC-PR), the Boltzmann-enhanced discrimination of the ROC curve (BEDROC), balanced accuracy, and the Matthews correlation coefficient (MCC).

##### Results

**Figure 4:**
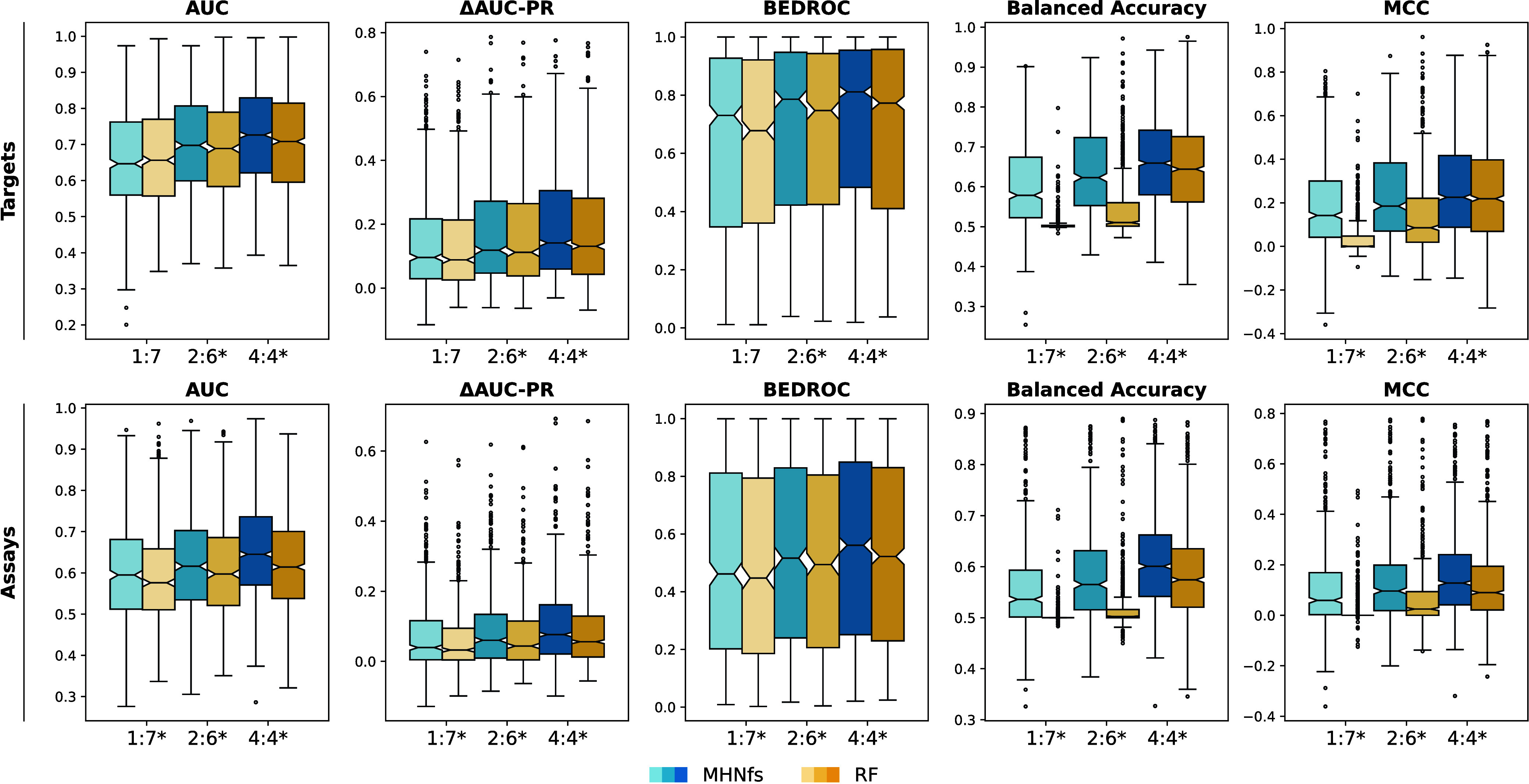
Results on the PubChem-based few-shot experiment with **support-set size 8**. Variation is shown across tasks and three draws of support sets. *n*:*m* indicate a support set with *n* active and *m* inactive molecules. Significantly different performances between the two models are indicated by a star.

**Figure 5:**
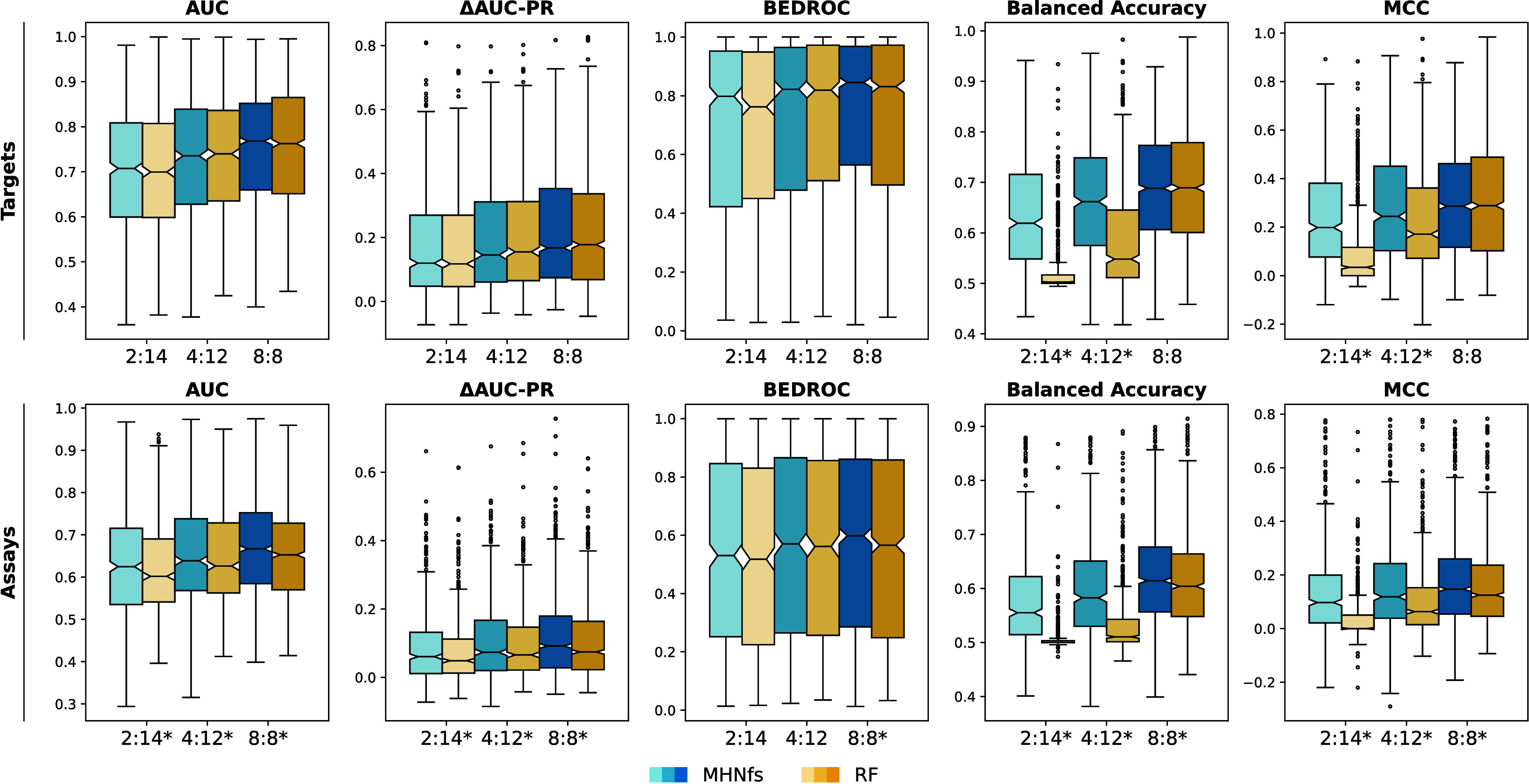
Results on the PubChem-based few-shot experiment with **support-set size 16**. Variation is shown across tasks and three draws of support sets. *n*:*m* indicate a support set with *n* active and *m* inactive molecules. Significantly different performances between the two models are indicated by a star.

The results with respect to the different metrics are presented in Figure 4 and Figure 5 for support-set sizes of 8 and 16. The variation of the metrics is shown across tasks and three draws of support sets. The stars on the *x*-axis labels indicate whether one method significantly outperforms the other (based on a two-sided paired Wilcoxon test). For almost all scenarios and metrics tested, MHNfs significantly outperforms the Random Forest baseline. The median ROC-AUC of MHNfs for targets given only 4 active and 4 inactive molecules, is already above 0.7, which should lead to enrichment of actives in wet-lab testing^64,65^ (Figure 4, top left panel).

## Discussion and Conclusions

In this work, we have introduced an application called *MHNfs*, which provides a simple and accessible user interface to a trained few-shot learning method for bioactivity prediction. With the *MHNfs* application, users can obtain relatively accurate activity predictions for their compound libraries, even if they know as few as one active and one inactive molecule. We validated the application on 229 activity prediction tasks from PubChem, using them to simulate few-shot learning scenarios. The straightforward design, together with the usability and predictive quality of the model, makes few-shot learning broadly accessible to practitioners and researchers beyond experts in chemoinformatics and computer-aided drug discovery. Based on the good performance at very small support sets, e.g., 4 actives and inactives, researchers in the field could be encouraged to start wet-lab screening of molecules early in projects when only scarce information is available. We hope that our work sparks investigations in the design of a context set, whose influence and design has not been thoroughly investigated. Also, for larger support-set sizes, MHNfs has shown promise^42^ and can be used without the need to set up model training. However, users should be aware that, as the number of known molecules increases, traditional methods such as RFs may perform better, requiring only moderate additional resources for model training. Snyder et al.^43^ report that from 50 measures molecules upward, classic machine learning methods start outperforming few-shot learning methods. We envision that *MHNfs* will become a widely used tool in early stage projects around small molecules and their bioactivities.

### Limitations and Recommendations

Few-shot models are known to have limited ability to generalize to unseen domains and to maintain predictive power under domain shifts.^37,42^ Therefore, we recommend using this application primarily for protein families—oxidoreductases, kinases, hydrolases, lysases, isomerases, ligases, and translocases—in which MHNfs was specifically evaluated and show good performance. For other targets, users should verify that similar proteins are represented in the FS-Mol training and evaluation sets (see https://github.com/microsoft/FS-Mol/blob/main/datasets/targets/target_info.csv).

## Data Availability

The HuggingFace application *MHNfs* is available here: https://huggingface.co/spaces/ml-jku/mhnfs. Code and data to train and evaluate the backbone model MHNfs is provided at: https://github.com/ml-jku/MHNfs.
